# PXR Functionally Interacts with NF-κB and AP-1 to Downregulate the Inflammation-Induced Expression of Chemokine CXCL2 in Mice

**DOI:** 10.3390/cells9102296

**Published:** 2020-10-15

**Authors:** Maya Okamura, Ryota Shizu, Taiki Abe, Susumu Kodama, Takuomi Hosaka, Takamitsu Sasaki, Kouichi Yoshinari

**Affiliations:** 1 Laboratory of Molecular Toxicology, School of Pharmaceutical Sciences, University of Shizuoka, 52-1 Yada, Suruga-ku, Shizuoka 422-8526, Japan; gp1537@u-shizuoka-ken.ac.jp (M.O.); r_shizu@u-shizuoka-ken.ac.jp (R.S.); abet@med.tohoku.ac.jp (T.A.); hosaka@u-shizuoka-ken.ac.jp (T.H.); t-sasaki@u-shizuoka-ken.ac.jp (T.S.); 2 Laboratory of Health Chemistry, Graduate School of Pharmaceutical Sciences, Tohoku University, 6-3 Aramaki-aoba, Aoba-ku, Sendai, Miyagi 980-8578, Japan; 3 Laboratory of Toxicology, Division of Pharmaceutical Sciences, Graduate School of Medicine, Dentistry and Pharmaceutical Sciences, Okayama University, 1-1-1 Tsushima-naka, Kita-ku, Okayama 700-8530, Japan; s-kodama@okayama-u.ac.jp

**Keywords:** PXR, NF-κB, AP-1, chemokine, CXCL2, nuclear receptor, liver injury model, anti-inflammation, gene regulation

## Abstract

Pregnane X receptor (PXR) is a liver-enriched xenobiotic-responsive transcription factor. Although recent studies suggest that PXR shows anti-inflammatory effects by suppressing nuclear factor kappa B (NF-κB), the detailed mechanism remains unclear. In this study, we aimed to elucidate this mechanism. Mice were treated intraperitoneally with the PXR agonist pregnenolone 16α-carbonitrile (PCN) and/or carbon tetrachloride (CCl_4_). Liver injury was evaluated, and hepatic mRNA levels were determined via quantitative reverse transcription polymerase chain reaction. Reporter assays with wild-type and mutated mouse *Cxcl2* promoter-containing reporter plasmids were conducted in 293T cells. Results showed that the hepatic expression of inflammation-related genes was upregulated in CCl_4_-treated mice, and PCN treatment repressed the induced expression of chemokine-encoding *Ccl2* and *Cxcl2* among the genes investigated. Consistently, PCN treatment suppressed the increased plasma transaminase activity and neutrophil infiltration in the liver. In reporter assays, tumor necrosis factor-α-induced *Cxcl2* expression was suppressed by PXR. Although an NF-κB inhibitor or the mutation of an NF-κB-binding motif partly reduced PXR-dependent suppression, the mutation of both NF-κB and activator protein 1 (AP-1) sites abolished it. Consistently, AP-1-dependent gene transcription was suppressed by PXR with a construct containing AP-1 binding motifs. In conclusion, the present results suggest that PXR exerts anti-inflammatory effects by suppressing both NF-κB- and AP-1-dependent chemokine expression in mouse liver.

## 1. Introduction

The nuclear receptor pregnane X receptor (PXR, also known as NR1I2) is a xenobiotic-sensing transcription factor. It is highly expressed in the liver and is activated by a large number of xenobiotics. Xenobiotic-activated PXR plays a crucial role in the induction of xenobiotic metabolism and excretion by regulating the gene expression of drug-metabolizing enzymes and drug transporters [1]. Thus, PXR protects our body via detoxification of harmful chemicals.

Recent studies have expounded the biological roles of PXR in the liver and intestines. PXR is associated with the regulation of hepatic energy metabolism through crosstalk with insulin response transcription factors, such as forkhead box protein O1 (FOXO1), cAMP response element-binding protein (CREB), and serine/threonine-protein kinase 2 (SGK2) [2,3,4]. PXR has been also reported to regulate the sensitivity of hepatocytes to proliferating signals by interacting with FOXO3 and yes-associated protein (YAP) [5,6,7,8,9]. Additionally, PXR is reported to regulate inflammatory signals.

PXR activation downregulated the expression of inflammation-related genes which were strongly induced by treatment with tumor necrosis factor-α (TNF-α) and lipopolysaccharide (LPS) in mouse primary hepatocytes and cultured cell lines [10,11,12]. These studies have suggested a mechanism of the PXR-mediated regulation of inflammation, where PXR interacts with nuclear factor kappa B (NF-κB), a major transcription factor for inflammatory signals, and inhibits its function to downregulate the target gene expression, such as TNF-α, interleukin-1β (IL-1β), and nitric oxide synthase 2 (NOS2), under LPS or TNF-α treatment [10,11]. Further, reporter gene assays with NF-κB binding motifs suggested that NF-κB-dependent gene transcription was prevented by human PXR with its ligand, rifampicin, in a dose-dependent manner [13]. Recently, we reported that the activation of PXR by pregnenolone 16α-carbonitrile (PCN) prevented the induced expression of *Nos2*, *Ccl2*, and *Cxcl2*, but not *Tnfa* and *Il1b*, in mouse liver at 3 h after concanavalin A treatment [14]. The differences in the regulation among the genes may be due to differences in their promoter contexts, such as transcription factor binding motifs, and inflammation-related transcription factors involved, apart from NF-κB.

The expression of inflammation-related genes is also regulated by the transcription factor activator protein 1 (AP-1), which is involved in multiple cellular events, such as differentiation, proliferation, and apoptosis upon various stimuli, including cytokines, growth factors, and viral infections [15]. NF-κB and AP-1 work in concert with each other to respond to immune and inflammatory signals. Several members of the nuclear receptor superfamily, such as glucocorticoid receptor (GR), peroxisome proliferator-activated receptors (PPARs), and liver X receptor (LXR), have been shown to modulate the immune system by interacting with both NF-κB and AP-1 [16].

In this study, we investigated the detailed molecular mechanism of the PXR-dependent suppression of inflammation in the liver using a carbon tetrachloride (CCl_4_)-induced liver injury model. With the findings from in vivo studies, we focused on the effect of PXR on the gene transcription of the chemokine *Cxcl2* via NF-κB and AP-1. Here, we hypothesized that PXR might be involved in a crosstalk with both NF-κB and AP-1 for attenuation of *Cxcl2* expression and, consequently, inflammation in the liver.

## 2. Materials and Methods

### 2.1. Materials

PCN and rifampicin were purchased from Sigma-Aldrich (St. Louis, MO, USA). TNF-α was purchased from Pepro Tech (Rocky Hill, NJ, USA). CCl_4_, phorbol 12-myristate 13-acetate (PMA), and BAY11-7082 were purchased from FUJIFILM Wako Pure Chemical (Osaka, Japan). Amprenavir was purchased from Combi-Blocks (San Diego, CA, USA). Oligonucleotides were commercially synthesized by Fasmac (Atsugi, Japan). All other reagents were obtained from FUJIFILM Wako Pure Chemical or Sigma-Aldrich, unless otherwise indicated.

### 2.2. Plasmid Preparation

p3A4-pGL3 plasmid and mouse and human PXR expression plasmids (mPXR-pTargeT and hPXR-pTargeT, respectively) were constructed previously [17]. phRL-TK was obtained from Promega (Madison, WI, USA). GR-interacting protein 1 (GRIP1)-expressing pFN21A plasmid and control pFN21A were purchased from Promega. NFκB-luciferase (NFκB-Luc) and AP1-Luc reporter constructs as well as control pTA-Luc reporter plasmid were purchased from Signosis (Sunnyvale, CA, USA). *Cxcl2*-Luc and *Ccl2*-Luc were prepared by inserting mouse *Cxcl2* promoter (−581 to −3) or *Ccl2* promoter DNA (−2519 to −68), which was amplified via PCR using KOD-FX (TOYOBO, Osaka, Japan) and mouse genomic DNA as a template, into the XhoI and HindIII sites or NheI and XhoI sites of pGL4.10 (Promega), respectively. Mutations were introduced using the KOD Plus mutagenesis kit (TOYOBO) with specific primer sets.

### 2.3. Animal Experiments

Male C57BL/6N mice (approximately 7 weeks old; Charles River Laboratories Japan, Yokohama, Japan) were maintained under a 12 h light/ 12 h dark cycle, fed with a conventional CE-2 laboratory diet (CLEA Japan, Tokyo, Japan), and were provided with water ad libitum for 1 week for acclimatization. The mice were then intraperitoneally administered with PCN (100 mg/kg), followed 6 h later by CCl_4_ (0.5 mL/kg, 10% v/v, in corn oil). After 24 h, they were sacrificed, and the blood and liver samples were collected. Plasma alanine aminotransferase (ALT) activity was determined using the Transaminase CII-B-test Wako (FUJIFILM Wako Pure Chemical) following the manufacturer’s instructions. All animal experiments were approved by the committee for animal experiments at University of Shizuoka and conducted in accordance with the guidelines for animal experiments at University of Shizuoka.

### 2.4. Cell Culture

Briefly, 293T cells (RIKEN BioResource Center, Tsukuba, Japan) were cultured in the Dulbecco’s modified Eagle’s medium (DMEM) (FUJIFILM Wako Pure Chemical) supplemented with heat-inactivated 10% fetal bovine serum (GE Healthcare, Buckinghamshire, UK), nonessential amino acids (Thermo Fisher Scientific, Waltham, MA, USA), and an antibiotic-antimycotic solution (100 U/mL penicillin, 100 μg/mL streptomycin, and 0.25 μg/mL amphotericin B; Thermo Fisher Scientific). The cells were seeded in 96-well plates (BD Biosciences, Heidelberg, Germany) at a density of 1 × 10^4^ cells/well. At 24 h after the seeding, the cells were transfected with plasmids.

### 2.5. Liver Histology Analysis

The left lobe of the liver was preserved in 10% neutral buffered formalin (FUJIFILM Wako Pure Chemical) and embedded in paraffin wax. The paraffin-embedded samples were sectioned at a thickness of 4 μm and stained with hematoxylin and eosin or subjected to immunostaining with anti-myeloperoxidase (MPO) antibody by Morphotechnology (Sapporo, Japan). MPO-positive nuclei were counted in three randomly selected areas per section from each individual mouse, and the percentage of MPO-positive nuclei was calculated for each area.

### 2.6. Quantitative Reverse Transcription Polymerase Chain Reaction (qRT-PCR)

Total RNA was isolated using Sepasol RNA I (Nacalai Tesque, Kyoto, Japan). mRNA levels were measured as per methods described previously [17]. The sequences of the primers used for qRT-PCR are shown in Supplementary Table S1.

### 2.7. Reporter Assays

At 24 h after seeding, cells were co-transfected with reporter gene plasmid, expression plasmid, and *Renilla* luciferase-expressing plasmid using Lipofectamine 3000 (Invitrogen, Carlsbad, CA, USA), and then treated with vehicle (0.1% or 0.2% dimethyl sulfoxide, DMSO) or drugs in serum-free DMEM for 24 h. Reporter activity was measured using the Dual-Luciferase Reporter Assay System (Promega) following the manufacturer’s instructions. Firefly luciferase luminescence was normalized to *Renilla* luciferase luminescence.

### 2.8. Statistical Analysis

Statistical analyses were conducted using GraphPad Prism 7 (GraphPad Software, San Diego, CA, USA). Significance of differences was assessed using the Student’s *t*-test for comparison of data between two groups and using one-way analysis of variance (ANOVA) followed by Dunnett’s or Tukey–Kramer post-hoc tests for comparison of data between multiple groups. *p*-values of less than 0.05 were considered as statistically significant, and significant differences have been indicated by asterisks. The *p*-values were not used for testing the experimental hypotheses but were indicated to highlight the differences between the compared groups. All experiments were repeated at least twice to confirm the reproducibility. Sample sizes were specified prior to the experiments, and the number of experiments to confirm reproducibility was determined after the initial results were obtained.

## 3. Results

### 3.1. PCN Treatment Ameliorates Liver Injury-Induced Expression of Chemokines in Mice

**Figure 1:**
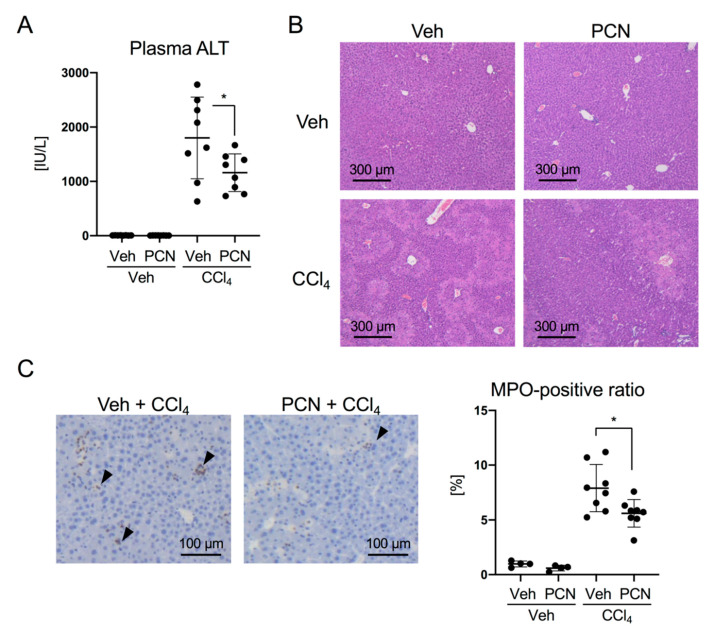
Effect of pregnenolone 16α-carbonitrile (PCN) treatment on carbon tetrachloride (CCl_4_)-induced liver injury. Mice were intraperitoneally treated with vehicle (Veh, corn oil) or PCN (100 mg/kg), and 6 h later treated with vehicle (Veh, corn oil) or CCl_4_ (0.5 mL/kg). After another 24 h, the mice were sacrificed. (**A**) Plasma alanine aminotransferase (ALT) activity was determined. Data are presented as the mean ± standard deviation (SD) with the plots of individual mouse data (*n* = 8). * *p* < 0.05 (Student’s *t*-test). (**B**) Liver sections were subjected to hematoxylin and eosin staining. Representative images are shown. (**C**) Liver sections were subjected to myeloperoxidase (MPO) staining. Arrowheads indicate MPO-positive nuclei. Ratios of MPO-positive nuclei to the total nuclei were calculated and are shown as relative values with that of the vehicle-treated mice as 1. Data are presented as mean ± SD with the plots of individual mouse data (*n* = 4–8). * *p* < 0.05 (Student’s *t*-test).

To investigate the anti-inflammatory effects of PXR, we first utilized CCl_4_-induced liver injury model mice. C57BL/6N mice were intraperitoneally pretreated with PCN, a mouse PXR (mPXR) agonist, and then with CCl_4_. As shown in Figure 1A, plasma ALT levels were substantially increased by CCl_4_ treatment, and this increase was significantly suppressed by PCN pretreatment. Consistent with these changes, focal areas of necrosis were reduced in the PCN/CCl_4_ group compared with those in the Veh/CCl_4_ group (Figure 1B). MPO staining showed that PCN pretreatment also reduced neutrophil infiltration in the liver (Figure 1C).

**Figure 2:**
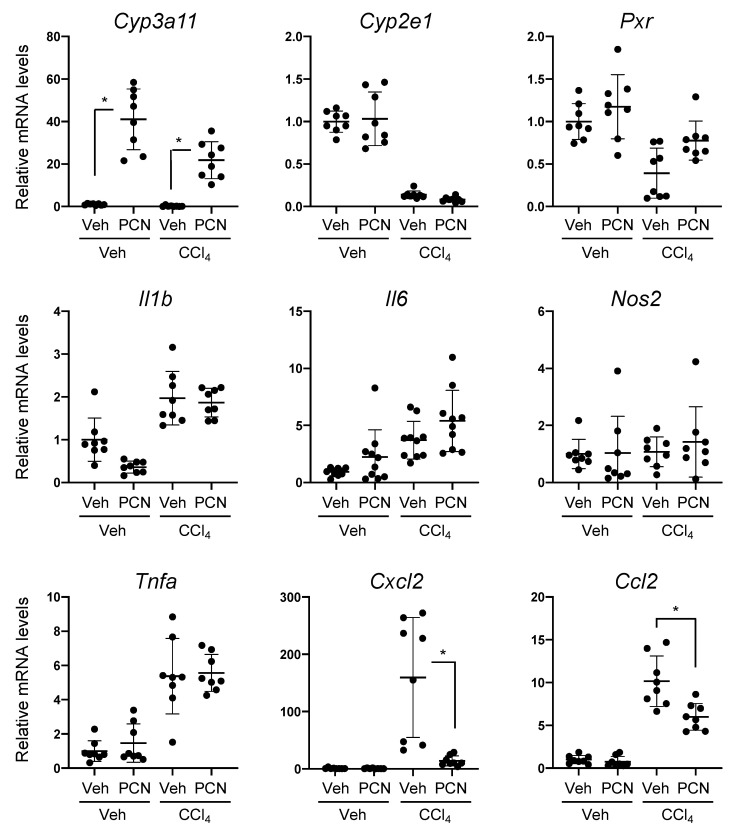
Effect of PCN treatment on the hepatic mRNA levels in CCl_4_-treated mouse. The total RNA from the mouse livers illustrated in Figure 1 was subjected to quantitative reverse transcription polymerase chain reaction (qRT-PCR). The mRNA levels of the target genes were normalized to 18S rRNA levels and are shown as relative values with that of the vehicle-treated mice as 1. Data are presented as mean ± SD with the plots of individual mouse data (*n* = 8). * *p* < 0.05 (Student’s *t*-test).

To investigate whether PXR activation affected the expression of proinflammatory genes and PXR target genes, qRT-PCR analyses were performed using the hepatic RNA prepared from the mice (Figure 2). As expected, PCN treatment strongly upregulated the mRNA levels of *Cyp3a11*, a representative mPXR target gene. The mRNA levels of *Pxr* and *Cyp2e1*, the latter of which encodes CYP2E1 that is involved in the metabolic activation of CCl_4_, were not affected by PCN treatment, but were reduced by CCl_4_ treatment. In this model, CCl_4_ treatment upregulated the mRNA levels of proinflammatory genes, including *Il6*, *Tnfa*, *Ccl2*, and *Cxcl2*. Moreover, the upregulation of *Ccl2* and *Cxcl2*, which encode chemokines, was suppressed by PCN pretreatment, whereas the upregulation of *Il6* and *Tnfa* was not affected by the treatment. These results suggest that PXR activation with PCN downregulates chemokine expression and ameliorates CCl_4_-induced liver injury in mice.

To confirm the suppressive effects of PXR on *Cxcl2* and *Ccl2* expression, we assessed the effects of another mPXR activator, amprenavir [18]. C57BL/6N mice were intraperitoneally pretreated with amprenavir and then treated with CCl_4_. At 3 or 24 h after CCl_4_ treatment, the mice were sacrificed, and hepatic mRNA levels were determined. Amprenavir treatment significantly upregulated *Cyp3a11* mRNA levels (Supplementary Figure S1A). CCl_4_ treatment upregulated the mRNA levels of *Ccl2* and *Cxcl2* within 3 h, whereas amprenavir pretreatment tended to suppress their levels. Amprenavir treatment-dependent suppression was clearly observed for *Cxcl2* mRNA levels but not for *Ccl2* mRNA levels at 24 h after CCl_4_ treatment (Supplementary Figure S1B).

### 3.2. PXR Suppresses the NF-κB-Dependent Cxcl2 Expression

**Figure 3:**
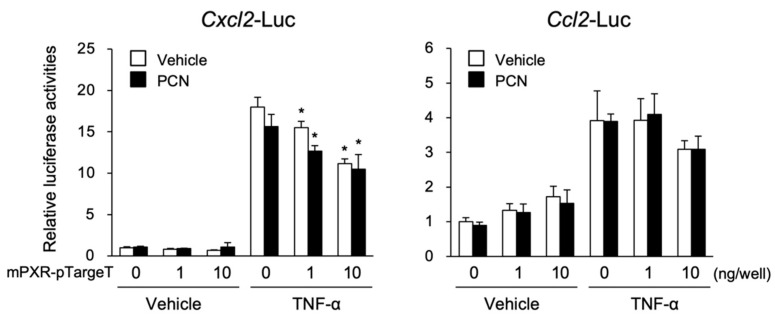
The influence of pregnane X receptor (PXR) activation on *Ccl2* and *Cxcl2* expression. 293T cells were transfected with *Cxcl2*-Luc or *Ccl2*-Luc, mPXR-pTargeT or control pTargeT, and control phRL-TK (total DNA: 100 ng/well) and then treated with 10 µM PCN or vehicle (0.1% dimethyl sulfoxide, DMSO) in combination with or without 10 ng/mL tumor necrosis factor-α (TNF-α) or vehicle (0.1% DMSO) for 24 h, and reporter activity was determined. Relative activity is shown compared to that in the cells transfected with control pTargeT (mPXR-pTargeT: 0 ng) and treated with vehicle only (the left-end open bar) as 1. Values are presented as mean ± SD (*n* = 4). Statistical differences within the TNF-α-treated groups were analyzed using one-way analysis of variance (ANOVA) followed by Dunnett’s test with the vehicle-treated cells with no PXR expression (the far-left column) as a control group. * *p* < 0.05.

To investigate whether PXR directly regulated the transcription of the chemokine-encoding genes *Ccl2* and *Cxcl2*, reporter assays using plasmids containing a mouse *Ccl2* or *Cxcl2* promoter were performed with TNF-α treatment in 293T cells (Figure 3).

As expected, the *Cxcl2*-driven luciferase reporter activity was considerably increased by TNF-α treatment. This increase was significantly suppressed by mPXR expression and PCN treatment. Similarly, the *Ccl2*-driven reporter activity was increased by TNF-α treatment, and, unexpectedly, the reporter activity was also increased by mPXR expression, although the extent of this increase was much lower than that of TNF-α. Despite this unexpected increase, TNF-α-induced *Ccl2*-driven reporter activity was suppressed by mPXR expression (at the highest dose) with or without PCN treatment. Similar results were observed with human PXR (hPXR) for the *Cxcl2* and *Ccl2* reporter plasmids (data not shown).

**Figure 4:**
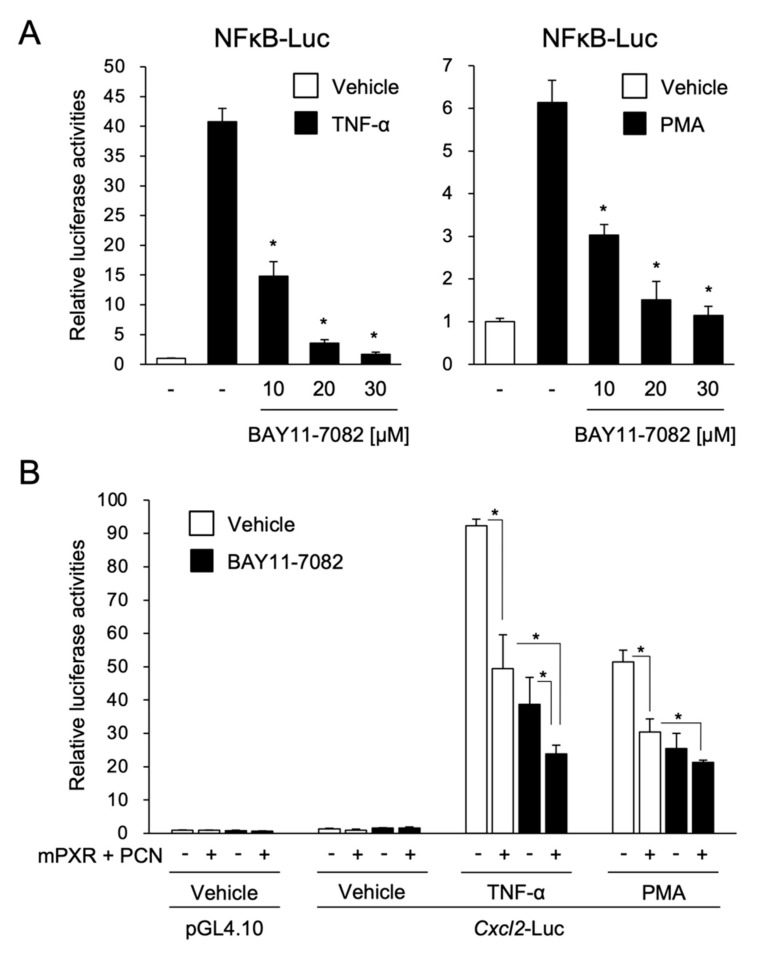
Effect of the nuclear factor kappa B (NF-κB) inhibitor and PXR activation on *Cxcl2* expression. (**A**) 293T cells were transfected with NFκB-Luc and control phRL-TK (total DNA: 100 ng/well). The cells were treated with 10 ng/mL TNF-α, 10 nM phorbol 12-myristate 13-acetate (PMA), or vehicle (0.1% DMSO) with or without 10, 20, or 30 µM BAY11-7082 for 24 h, and reporter activity was determined. Relative activity is shown compared to that in the cells treated with vehicle only (open bars) as 1. Values are presented as mean ± SD (*n* = 4). Statistical differences among the TNF-α-treated groups were analyzed using one-way ANOVA followed by the Dunnett’s test, with the cells without BAY11-7082 treatment as a control group (* *p* < 0.05). (**B**) 293T cells were transfected with *Cxcl2*-Luc or control pGL4.10, mPXR-pTargeT (10 ng) or control pTargeT, and control phRL-TK (total DNA: 100 ng/well). The cells were treated with 10 ng/mL TNF-α, 10 nM PMA, or vehicle (0.1% DMSO) in combination with or without 30 µM BAY11-7082 and 10 µM PCN for 24 h, and reporter activity was determined. Relative activity is shown compared to that in the cells transfected with pGL4.10 and control pTargeT and treated with vehicle only (the left-end open bar) as 1. Values are presented as mean ± SD (*n* = 4). Difference within the TNF-α- or PMA-treated groups were analyzed using one-way ANOVA followed by the Tukey–Kramer test (* *p* < 0.05).

Using the *Cxcl2* gene as a model, we next investigated the molecular mechanism of mPXR-mediated suppression. As several reports showed a mutual repression of PXR and NF-κB [19,20,21], we investigated whether mPXR suppressed the NF-κB-mediated transcription of *Cxcl2*. To this end, we first assessed NF-κB function in our reporter assay system. The assays were performed using a reporter plasmid containing NF-κB-responsive elements (Figure 4A). Reporter activity was upregulated by TNF-α or PMA treatment. This upregulation was suppressed by treatment with the NF-κB inhibitor BAY11-7082 in a dose-dependent manner. BAY11-7082 is known to inhibit NF-κB activation by inhibiting the phosphorylation of IκBα, NF-κB inhibitor α, by IκBα kinase and its degradation [22]. These results suggest that NF-κB is functional in our experimental system.

Next, assays were performed using the *Cxcl2* reporter plasmid. Both TNF-α and PMA treatment drastically increased the *Cxcl2*-driven reporter activity and this increase was suppressed by mPXR expression and PCN treatment (Figure 4B, column 1 vs. 2) as well as by BAY11-7082 treatment (Figure 4B, column 1 vs. 3). In the case of TNF-α stimulation, mPXR expression and PCN treatment further suppressed the reporter activity even in the presence of BAY11-7082 (Figure 4B, column 3 vs. 4). These results imply that not only NF-κB but also other factor(s) are involved in mPXR-dependent suppression of inflammatory signal-induced *Cxcl2* expression.

### 3.3. PXR Downregulates AP-1- and NF-κB-Dependent *Cxcl2* Expression

**Figure 5:**
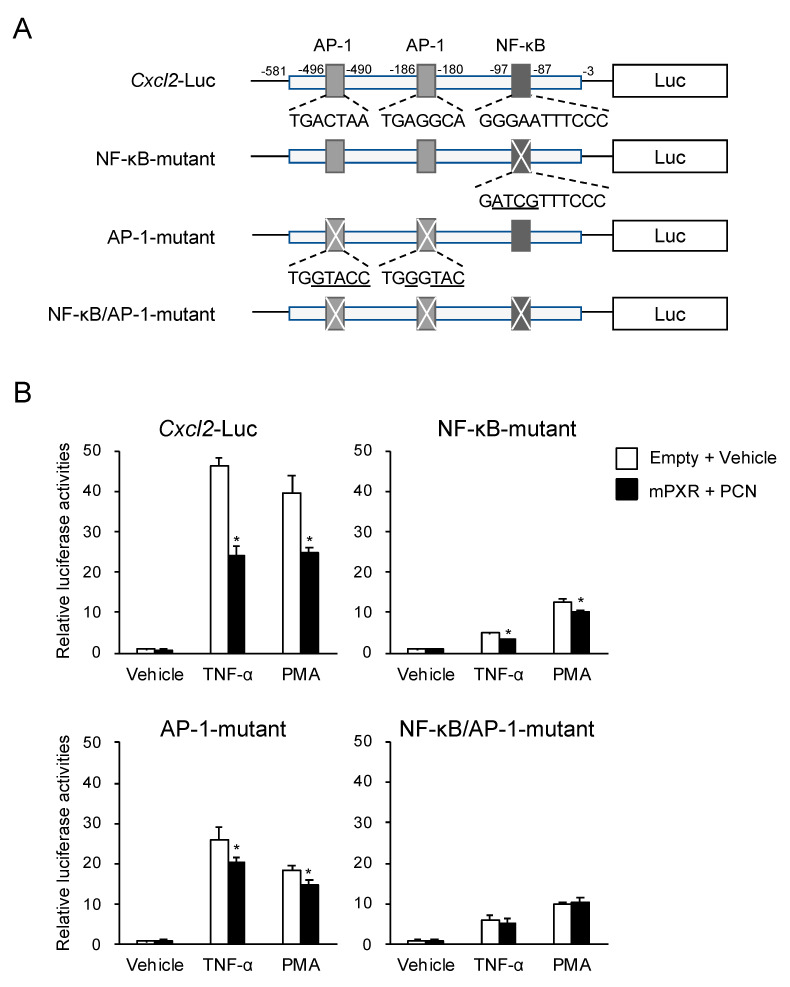
Effect of the mutation of NF-κB and activator protein 1 (AP-1) binding sites on the PXR-dependent suppression of *Cxcl2* expression. (**A**) Schematic representation of the reporter constructs is shown. Underlines indicate the mutated nucleotides. (**B**) 293T cells were transfected with each reporter plasmid shown in A, mPXR-pTargeT or control pTargeT, and control phRL-TK (total DNA: 100 ng/well). The cells were treated with 10 ng/mL TNF-α, 10 nM PMA, or vehicle (0.1% DMSO) in combination with or without 10 µM PCN or vehicle (0.1% DMSO) for 24 h, and reporter activity was determined. Relative activity is shown compared to that in the cells transfected with empty pTargeT and treated with vehicle only (the left-end open bar) as 1. Values are presented as mean ± SD (*n* = 4). * *p* < 0.05 (vs. “Empty + Vehicle”; Student’s *t*-test).

As *Cxcl2* expression is regulated by both NF-κB and AP-1 [23], we investigated whether PXR also downregulated AP-1-mediated *Cxcl2* expression. As shown in Figure 5A, the *Cxcl2* promoter contains two AP-1 binding motifs and one NF-κB binding motif [23]. Therefore, their binding motifs were mutated, and reporter assays were performed (Figure 5B). The *Cxcl2*-driven reporter activity was substantially upregulated by TNF-α or PMA treatment, and the upregulation was partly suppressed by mPXR expression with PCN treatment. The reporter activity of the NF-κB- or AP-1-mutated construct continued to exhibit upregulated expression by TNF-α or PMA treatment, and PCN-activated mPXR suppressed the increases, although the magnitude of TNF-α or PMA upregulation and mPXR suppression was lower than that of the wild-type construct. Moreover, when a reporter plasmid, in which all the NF-κB and AP-1 binding motifs were mutated, was used, TNF-α or PMA treatment continued to increase its reporter activity, but the increase was not suppressed by mPXR. These results suggest that mPXR downregulates *Cxcl2* expression by attenuating both NF-κB- and AP-1-dependent gene transcription.

**Figure 6:**
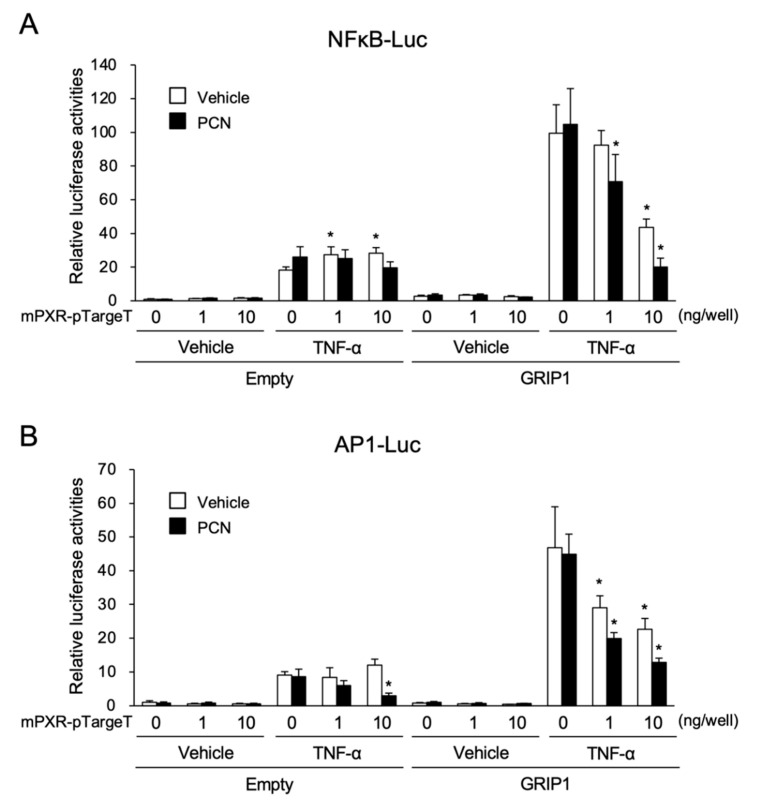
Effect of PXR on NF-κB- and AP-1-mediated gene transcription. 293T cells were transfected with NFκB-Luc (**A**) or AP1-Luc (**B**), mPXR and/or GRIP1 expression plasmid and/or their control plasmid (pTargeT and/or pFN21A), and control phRL-TK (total DNA: 100 ng/well). The cells were treated with 10 ng/mL TNF-α or 10 nM PMA in combination with or without 10 µM PCN or vehicle (0.1% DMSO) for 24 h, and reporter activity was determined. Relative activity is shown compared to that in the cells transfected with control plasmid and treated with vehicle only (the left-end open bar) as 1. Values are presented as mean ± SD (*n* = 4). Statistical differences within the TNF-α-treated groups were tested using one-way ANOVA followed by Dunnett’s test, with the vehicle-treated cells with no PXR expression (the far-left column) as a control group. * *p* < 0.05.

Finally, to further confirm the suppressive effects of ligand-activated PXR on the transactivation by NF-κB and AP-1, reporter assays were performed using a reporter construct containing consensus NF-κB (5′-GGGAATTTCC-3′) or AP-1 (5′-TGACTAA-3′) binding motifs (Figure 6A). As GRIP1, a transcriptional coactivator, was recently reported to enhance gene transcription by NF-κB or AP-1 [17], we expressed GRIP1 in this system (Figure 6A, right). With the reporter plasmid containing NF-κB binding motifs, the luciferase activity was increased via TNF-α treatment by approximately 18- and 35-fold in the absence and presence of GRIP1, respectively, and the increase was substantially suppressed by mPXR expression with PCN treatment in the presence of GRIP1. Similarly, the reporter activity of the construct with AP-1 binding motifs showed significant increases by PMA treatment, which was approximately 9- and 44-fold in the absence and presence of GRIP1, respectively, and the increase was considerably suppressed by mPXR expression and PCN treatment both with and without GRIP1. These results imply that mPXR can compete with NF-κB and AP-1 for GRIP1 binding and suppress their transcriptional activity.

## 4. Discussion

Recent studies have revealed that PXR plays a role in the regulation of inflammatory signals [20,21,24]. Several PXR-activating xenobiotics/drugs, such as rifampicin, phenytoin, or other natural products, have been reported to show immunosuppressive effects [25,26,27]. However, the details underlying PXR-mediated immunosuppression remain elusive. Several studies have shown that ligand-activated PXR exerts immunosuppressive and anti-inflammatory effects by antagonizing NF-κB [19,20,21]. This negative crosstalk between PXR and NF-κB is the only mechanism that has been reported to explain the effects of PXR thus far. However, our recent study has suggested that PXR-dependent immunosuppressive and anti-inflammatory effects are mediated by not only NF-kB but also other immune/inflammation-related transcription factor(s) in a concanavalin A-dependent liver injury model [14]. In this study, we attempted to identify the molecule(s) involved in PXR-mediated immunosuppression/anti-inflammation using in vivo and in vitro systems.

Using the CCl_4_-induced liver injury mouse model, we revealed that PCN pretreatment attenuated the degree of liver injury, based on plasma ALT activity and MPO staining of liver sections. Concomitantly, CCl_4_-induced expression of *Ccl2* and *Cxcl2* but not *Il6* and *Tnfa* in mouse livers were suppressed by PCN pretreatment. Reporter assays with the *Cxcl2* promoter showed that the PXR-dependent suppression was not completely abolished by treatment with the chemical inhibitor of NF-κB or the mutation of the NF-κB binding motif. These results corroborate the idea that a transcription factor(s) other than NF-κB contributes to the PXR-dependent suppression of *Cxcl2* expression.

AP-1 is known as a major immunoregulatory transcription factor that regulates a number of genes, including *Cxcl2* [15,22]. In our reporter assays, PXR-dependent suppression was observed with the *Cxcl2* reporter constructs containing the mutation of either NF-κB or AP-1 binding sites as well as the wild-type construct. However, when both the NF-κB and AP-1 binding sites were mutated, the PXR-dependent suppression disappeared even though the promoter continued to respond to TNF-α or PMA treatment. Additionally, in a reporter assay with constructs containing NF-κB or AP-1 binding motifs independent of *Cxcl2,* as shown in Figure 6, not only NF-κB- but also AP-1-dependent gene transcription induced by TNF-α or PMA was suppressed by PXR. These results strongly suggest that PXR attenuates both NF-κB and AP-1 signals in the regulation of *Cxcl2* expression.

It is well known that GR directly interacts with NF-κB and AP-1 to repress transcription mediated by NF-κB and AP-1, which is a key mechanism for the anti-inflammatory effects of glucocorticoids [28,29]. Additionally, RXRα, another member of the nuclear receptor superfamily, is reported to directly interact with NF-κB and inhibit its DNA binding in in vitro assays [19,30]. These previous findings indicate that the direct interaction of PXR with inflammation-related transcription factors is important for PXR-mediated immunosuppression.

In this regard, the PXR-mediated attenuation of NF-κB and AP-1 functions was clearly observed when their coactivator GRIP1 (also known as NCOA2) was co-expressed. Recently, we also reported that GRIP1, which is known to act as a PXR coactivator [31], augmented NF-κB- and AP-1-dependent gene expression, and PXR competes with NF-κB and AP-1 for GRIP1 interaction [17]. Na et al. also reported that the RXRα-dependent inhibition of NF-κB disappeared when the AF2 coactivator interaction domain was deleted from RXRα in reporter gene assays [30]. Moreover, SRC1 (also known as NCOA1) and ASC2 (also known as NCOA6) have been reported to act as coactivators for NF-κB- and AP-1-dependent as well as PXR-dependent gene transcription [31,32,33,34,35,36]. Based on these reports and our present findings, the PXR-dependent inhibition of NF-κB and AP-1 may involve coactivator competition or squelching.

## 5. Conclusions

We have shown that PXR activation inhibits inflammatory signal-induced mouse *Cxcl2* expression, and our studies on its molecular mechanism have identified AP-1 as a novel target of PXR for the downregulation of *Cxcl2* expression. Our present findings will help us uncover the mechanism underlying the anti-inflammatory effects of PXR and aid in the development of a new class of immunosuppressive drugs.

## Supplementary Materials

The following are available online at https://www.mdpi.com/2073-4409/9/10/2296/s1, Figure S1: Effect of amprenavir treatment on CCl_4_-dependent induction of the hepatic mRNA levels of *Ccl2* and *Cxcl2* in mice. Table S1: Primers used for qRT-PCR.
