# Supplementary material for: PXR Functionally Interacts with NF-κB and AP-1 to Downregulate the Inflammation-Induced Expression of Chemokine CXCL2 in Mice
Source: Cells. 2020 Oct 15;9(10):2296. doi: 10.3390/cells9102296 (PMC7602528; doi:10.3390/cells9102296)
Supplement: Supplementary file 1 [file cells-09-02296-s001.zip › FigureS1.pdf]

Figure S1

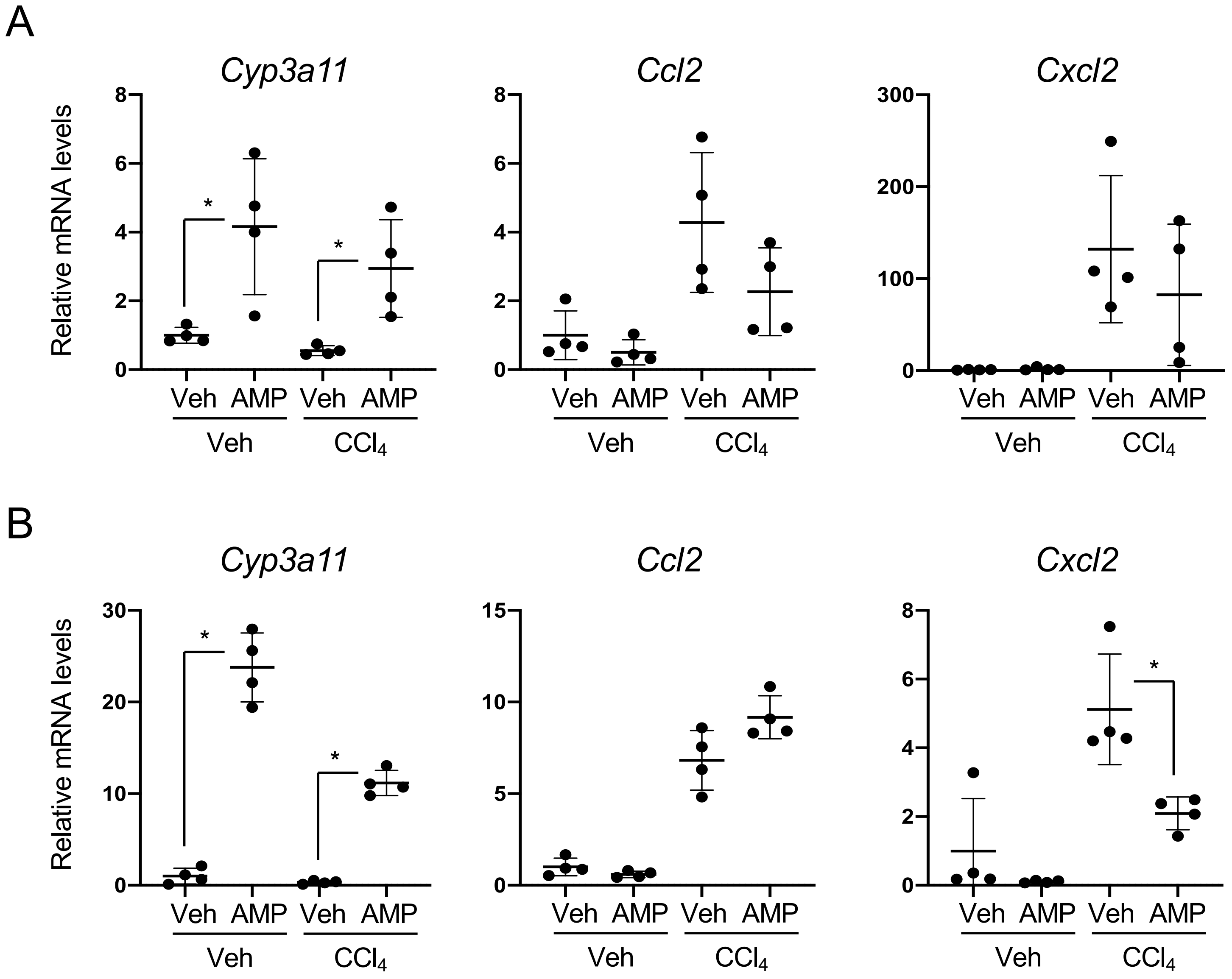

Figure S1. Effect of amprenavir treatment on CCl_4-dependent induction of the hepatic mRNA levels of *Ccl2* and *Cxcl2* in mice. Mice were intraperitoneally treated with vehicle (Veh, corn oil) or amprenavir (AMP, 100 mg/kg) and 6 h later with vehicle (Veh, corn oil) or CCl_4 (0.5 mL/kg). The mice were killed at 3 (A) or 24 h (B) after CCl_4 treatment. Total RNA from the livers was prepared and subjected to qRT-PCR. The mRNA levels of the target genes were normalized to 18S rRNA levels and are shown as relative values with that of the vehicle-treated mice as 1. Data are presented as mean ± S.D. with the plots of individual mouse data (n=4). $^*P < 0.05$ (Student's *t*-test).
