# Supplementary material for: PXR Functionally Interacts with NF-κB and AP-1 to Downregulate the Inflammation-Induced Expression of Chemokine CXCL2 in Mice
Source: Cells. 2020 Oct 15;9(10):2296. doi: 10.3390/cells9102296 (PMC7602528; doi:10.3390/cells9102296)
Supplement: Supplementary file 1 [file cells-09-02296-s001.zip › TableS1.docx]

**Table S1**. Primers used for qRT-PCR.

Gene Forward primer (5’ to 3’) Reverse primer (5’ to 3’) Amplification efficiency^*^

*Ccl2* CCACTCACCTGCTGCTACTCAT TGGTGATCCTCTTGTAGCTCTCC 106%

*Cxcl2* AAGTTTGCCTTGACCCTGAAG ATCAGGTACGATCCAGGCTTC 109%

*Cyp3a11* ACAAGCAGGGATGGACCTGG TGTGACAGCAAGGAGAGGCG 130%

*Cyp2e1* TCTGCAGGAAAGCGCGTGTGT TCGCGTGGGATACTGCCAAAGC 105%

*Il1b* GCAACTGTTCCTGAACTCAACT ATCTTTTGGGGTCCGTCAACT 102%

*Il6* TAGTCCTTCCTACCCCAATTTCC TTGGTCCTTAGCCACTCCTTC 99%

*Nos2* CACCTTGGAGTTCACCCAGT ACCACTCGTACTTGGGATGC 98%

*Pxr* GGTGTGGTCCAGCGCAGCGT ACTGCTGGGTTTGCTGGGCGT 104%

*Tnfa* CCCTCACACTCAGATCATCTTCT GCTACGACGTGGGCTACAG 97%

18s rRNA ACCGCGGTTCTATTTTGTTG AGTCGGCATCGTTTATGGTC 100%

^*^Amplification efficiency was determined using a series of diluted mouse liver cDNA for each primer set.
